# ASCO 2018 NSCLC highlights—combination therapy is key

**DOI:** 10.1007/s12254-018-0444-7

**Published:** 2018-10-22

**Authors:** Gabriele Gamerith, Florian Kocher, Jakob Rudzki, Andreas Pircher

**Affiliations:** Department of Internal Medicine V (Hematology and Oncology), Medical University of Innsbruck, Anichstraße 35, 6020 Innsbruck, Austria

**Keywords:** Lung cancer, Immunotherapy, Combination therapy, Anti-angiogenesis, NSCLC screening

## Abstract

Non-small cell lung cancer (NSCLC) treatment was booming at this year’s ASCO 2018 meeting as several well-performed phase III trials with practice-changing potential were presented. Thereby immune checkpoint blockade (ICB) consolidated its major role in the treatment of NSCLC patients without genetic alterations and extended its use by showing impressive data on ICB combination therapies (mainly combined with chemotherapy). Furthermore the role of predictive biomarkers for ICB therapy (Programmed death-ligand 1 [PD-L1] expression, tumor mutational burden [TMB] testing and others) have been further developed and blood-based tests were presented with promising data revealing the potential of this minimally invasive method for treatment monitoring and guidance in the future. Nevertheless the best biomarker is still elusive and future research is ongoing and might be a multimodal approach combining different modalities. No major studies concerning new genetic alterations or innovative targets were presented and the focus in genetic driven NSCLC was the evaluation of combinational approaches (e.g. in epidermal growth factor receptor [EGFR] mutation positve patients, EGFR tyrosine kinase inhibitor [TKI] plus anti-angiogenic agent or chemotherapy backbone). The presented results showed some benefit for the combinational approach; however toxicity might be an issue and further validation is necessary. Summarizing, ASCO 2018 showed that combinational approaches will be the future standard treatment in NSCLC and that biomarker identification is more heterogeneous and complex than anticipated, but presented next generation techniques may pave the way to a more personalized cancer therapy.

## Background

The therapeutic landscape of lung cancer is changing rapidly due to better characterization of non-small cell lung cancer (NSCLC) genetics and identification of hallmark immunobiological characteristics. At the ASCO 2018 meeting, however, no major advances regarding NSCLC genetics as well as druggable targets were presented. However, deep molecular testing was evaluated as a lung cancer-screening tool and preliminary data were presented. Concerning new therapeutic strategies many impressive results on the use of ICB in combination therapy or as ICB monotherapy in advanced stage NSCLC were presented establishing new future therapy standards. Some trials gave us a new view on future combinational approaches of targeted agents in genetic driven NSCLC, but these data are not yet of practical relevance. In the following a subjective selection of presentations is displayed and discussed.

## Lung cancer screening using cell-free DNA

The Circulating Cell-Free Genome Atlas (CCGA) study attempts to study highly sophisticated genetic techniques for cancer screening. Currently, the study has enrolled more than 12,000 of the planned 15,000 participants (70% with cancer, 30% without cancer), in the United States and Canada. At ASCO the results of the first preplanned substudy from the CCGA was presented in which three sequencing assays (targeted sequencing [TS], whole-genome sequencing [WGS], whole-genome bisulfite sequencing [WGBS]) were performed on blood samples [1]. The mentioned three techniques were analyzed in 127 patients with stage I–IV lung cancer. Among these the lung cancer signature on cell-free DNA (cfDNA) was comparable across the assays and the signal increased with cancer stage. At 98% specificity, the WGBS assay detected 41% of early stage (stage I–IIIA) lung cancers and 89% of late-stage (stage IIIB–IV) cancers. The other two assays—the WGS assay and the TS assay—showed similar sensitivity in detecting early and late-stage disease. However, wide confidence intervals indicate the need for fine-tuning and limitations of these methods in individual cases. Nevertheless, this study showed that cfDNA-based assays are feasible and generated high quality, reproducible and comparable data, but clinical validation is still warranted. In addition, the used techniques pose methodological challenges by providing enormous amounts of data that require complex bioinformatic processing and further validation (studies are already ongoing).

## First-line IO therapy versus chemotherapy alone

In the ASCO’s plenary session the KEYNOTE-042 trial was presented, a phase 3 study in NSCLC patients with proven PD-L1 expression greater than 1% (subgroups: tumor proportion score [TPS] PD-L1 ≥1% or greater, ≥20%, and ≥50%), assessing the use of pembrolizumab 200 mg every 3 weeks versus investigator’s choice chemotherapy (platinum doublet therapy). Overall survival (OS), the primary end point of the study, was improved across subgroups [2]. Progression-free survival (PFS) was not positive at this interim analysis. The duration of response with pembrolizumab was improved in all subgroups compared to chemotherapeutic treatment.

In the TPS ≥50% subset, median OS was 20 months (range 15.4–24.9) in the pembrolizumab-treated patients and 12.2 months in those treated with chemotherapy (hazard ratio [HR] 0.69; 95% CI, 0.56–0.85; *P* = 0.0003). Similarly, in the TPS ≥20% subset, median OS was 17.7 months in the pembrolizumab-treated patients and 13.0 months in those treated with chemotherapy (HR 0.77; 95% CI, 0.64–0.92; *P* = 0.0020). In the whole study population (i.e. TPS ≥1%), median OS was 16.7 months in the pembrolizumab-treated and 12.1 months in those patients treated with chemotherapy (HR 0.81; 95% CI, 0.71–0.93; *P* = 0.0018). An exploratory subgroup analysis was conducted in patients with a TPS of 1–49%, which showed an OS HR of 0.92 (95% CI, 0.77–1.11). Concluding that the overall positive study results were significantly influenced by the population of the PD-L1 high expressing patients (about 50% of included patients). Therefore, the results are mainly confirmatory of the previous findings of the KEYNOTE-024 study [3]; however the study opens, due to a more favorable toxicity profile (grade 3 to 5 adverse events 17.8% vs 41.0%) in PD-L1 1–49% patients a new treatment option with pembrolizumab monotherapy. However this strategy has to be evaluated carefully as several new combinational trials have been presented and will be summarized in the following. Furthermore it has to be clearly stated that the PD-L1 high expressers generated the overall study benefit and that the study did not allow crossover further influencing the OS interpretation.

## First-line IO combination with chemotherapy versus chemotherapy alone

Four important phase III trials investigating the role of ICB combination therapy versus chemotherapy or ICB/ICB therapy were presented (most important endpoints are summarized in Table 1) and each study will be shortly summarized in the following [4–7].

**Table 1:** Summary of main results of phase III ICB + chemotherapy trials presented at the 2018 ASCO annual meeting

Trial	IMPower150		IMPower131		KEYNOTE-407		CHECKMATE 227		
*n*	1202		1021		559		129		
*Selection*	Nonsquamous		Squamous		Squamous		PD-L1 <1%, TMB ≥10 mut/Mb		
*Study Arms*	Atezolizumab+ bevacizumab+ carboplatin/paclitaxel	Bevacizumab+ carboplatin/ paclitaxel	Atezolizumab+ carboplatin/ nab-paclitaxel	Carboplatin/ nab-paclitaxel	Pembrolizumab+ carboplatin/ paclitaxel or nab-paclitaxel	Carboplatin/ paclitaxel or nab-paclitaxel	Nivolumab+ ipilimumab	Nivolumab+ chemotherapy	Chemotherapy
*ORR*	63.5%	48.0%	59.4%	51.3%	58.4%	35.0%	36.8%	60.5%	20.8%
*DOR*	9.0 months	5.7 months	6.6 months	4.4 months	7.7 months	4.8 months	NR	7.4 months	4.4 months
*PFS*	8.3 months	6.8 months	6.3 months	5.6 months	6.4 months	4.8 months	7.7 months	6.2 months	5.3 months
*OS*	19.2 months	14.7 months	14.0 months	13.9 months	15.9 months	11.3 months	NA	NA	NA
*Grade 3–5 Toxicity*	58.5%	50.0%	68%	57%	69.8%	68.2%	26%^a^	53%^a^	36%^a^

*n* number, *ORR* overall response rate, *PFS* progression-free survival, *OS* overall survival, *NR* not reached, *NA* not available, *ICB* immune checkpoint blockade, *PD-L1* programmed death-ligand 1, *DOR* duration of response, *TMB* tumor mutational burden, *mut/MB* mutations per megabase

^a^Grade 3–5 Toxicity in whole study collective

The OS data of the IMpower150 study (only non-squamous NSCLC) were presented as late breaking abstract. The study was designed to assess whether the quadruple therapy with the addition of atezolizumab (anti-PD-L1 antibody) to a backbone of carboplatin, paclitaxel, and bevacizumab (ABCP) is superior to the triple therapy with BCP alone [5]. The study included also a third comparison arm investigating a chemotherapy backbone plus atezolizumab (for further study details we refer to the full text publication [8]). For the first time this study included specific subgroups of patients with ALK translocations and activating EGFR mutations (only patients with disease progression or with unacceptable side effects from treatment with at least one approved tyrosine kinase inhibitor were included). In the complete intention to treat (ITT) population the median OS of ABCP treated patients was 19.8 months compared with 14.9 months in the BCP arm (HR, 0.76; 95% CI, 0.63–0.93). The 24-month OS rate was 45% with ABCP compared to 36% with BCP. ABCP also improved median progression-free survival (PFS) by 1.5 months compared with BCP (8.3 vs 6.8 months; HR, 0.59 in the ITT wildtype population). According to subgroup analysis especially patients with genetic alterations or liver metastasis seemed to benefit from the ABCP combination therapy, concluding that the combination of chemotherapy, anti-angiogenic therapy and ICB-therapy is highly effective, particular in certain patient subgroups. Nevertheless, toxicity was also increased, even though tolerable (see Table 1). It has to be considered that these subgroup analyses were not preplanned; however presence of liver metastasis was a stratification factor (besides PD-L1 expression and sex).

For advanced squamous NSCLC novel therapeutic options are also of high medical need and at ASCO 2018 two promising phase III studies (IMpower131 and KEYNOTE-407) have been presented testing chemotherapy backbone combined with IO therapy [4, 6]. The IMpower131 was a study in treatment-naive patients with squamous NSCLC. Regardless of their level of PD-L1 expression the patients were randomized to receive carboplatin and paclitaxel plus atezolizumab (arm A), carboplatin and nab-paclitaxel plus atezolizumab (arm B) and carboplatin and nab-paclitaxel (arm C). The primary endpoints of the study were investigator-assessed PFS and OS. Data showed a doubling in PFS at 12 months from 12% in the control arm to 24.7% in the atezolizumab+carboplatin/nab-paclitaxel arm translating in a PFS of 6.3 months versus 5.6 months. The response rates increased in the PD-L1-high category from 33 to 60%, and a significant improvement of the duration of response (median DOR 18.7 vs 5.3 months) was observed. However first OS data were not positive and Kaplan–Meier curves started to divide at 20 months (further analysis are needed and expected at the end of 2018). The second combination of chemotherapy plus IO therapy trial was KEYNOTE-407 (carboplatin and paclitaxel/nab-paclitaxel ± pembrolizumab). In this study OS was significantly improved in the pembrolizumab-containing arm: median OS of 15.9 months vs 11.3 months in the chemotherapy arm (HR 0.64, 95% CI [0.49, 0.85]; *p* = 0.0008). In all predefined subgroups OS was superior in the pembrolizumab/chemotherapy arm. The magnitude of the survival benefit from pembrolizumab added to chemotherapy was similar among all PD-L1 subgroups. PFS also favored the combination of pembrolizumab/chemotherapy compared to chemotherapy (median PFS 6.4 vs. 4.8 months; HR 0.56, 95% CI [0.45, 0.70]; *p* < 0.0001). PFS was better with the addition of pembrolizumab in all three PD-L1 TPS categories, but the reduction on the rate of disease progression correlated with the PD-L1 expression.

Regarding new biomarker development and efficacy of ICB combination therapy in PD-L1 negative patients (ICB/chemotherapy or ICB/ICB) results from the Checkmate 227 study were presented [7]. This study also intended to investigate tumor mutational burden (TMB) as a biomarker for ICB efficacy. The study showed that nivolumab combined with chemotherapy significantly prolonged PFS compared to chemotherapy alone in PD-L1 negative patients (5.6 months versus 4.7 months, HR 0.74, 95% CI [0.58, 0.94]). Subgroup analysis revealed that patients with high TMB benefitted most from nivolumab chemotherapy combination (around 50% of treated patients were evaluable for TMB testing). Next, patients were stratified according to TMB low and high status. In these populations of patients the IO combination treatment of nivolumab plus ipilimumab was compared with the two other treatment arms (for detailed study description we refer to the original publication [9]). Summarizing the results, the ICB/ICB combination was most effective in TMB high/PD-L1 negative patients followed by nivolumab plus chemotherapy and chemotherapy alone. In the TMB low/PD-L1 negative setting all three treatment modalities were similarly effective and nivolumab plus chemotherapy or nivolumab plus ipilimumab did not improve the outcome of the patients compared to chemotherapy alone. The study showed that TMB is a good predictive biomarker for the combination of nivolumab/chemotherapy and nivolumab/ipilimumab. According to the results, TMB low and PD-L1 negative patients do not seem to benefit from ICB therapy and chemotherapy remains the standard of care. However for broad clinical use TMB testing has to be harmonized and included in routine work-up and the data have to be validated prospectively. Furthermore, at the moment it is not clear if the PFS benefit translates into an OS benefit. Therefore the OS data are highly awaited.

## ICB combination therapy in higher therapy lines

An interesting study (phase II) was presented by a Dutch study group validating the so-called abscopal effect [10]. The study investigated the efficacy and safety of pembrolizumab after stereotactic body radiotherapy (SBRT) compared with pembrolizumab alone in patients with advanced NSCLC beyond second line therapy (PEMBRO-RT study [10]). Interestingly, this small phase II study could show that PFS was significantly prolonged in the pembrolizumab–radiotherapy (RT) arm compared to pembrolizumab alone (median PFS 1.8 months in the control arm vs 6.4 months in the experimental arm; HR 0.55; 95% CI [0.31, 0.98], *p* = 0.04). The OS benefit was not statistically significant but a trend towards improved survival was observed (median OS 19.2 months vs. 7.6 months, HR 0.58). The most common adverse events were fatigue, nausea, fever and hypothyroidism. No increase in treatment-related toxicity was observed in the experimental arm. Exploratory subgroup analysis implied that the benefit of SBRT given prior to pembrolizumab might be predominantly in patients with PD-L1 negative tumors. This interesting approach to achieve immunostimulation by RT has to be further investigated in larger phase III trials and results are much anticipated.

## IO therapy in genetic driven NSCLC

The clinical use of ICB in genetic driven NSCLC was retrospectively studied by Mazieres and colleagues and presented at the meeting [11]. The so-called ImmunoTarget study investigated the efficacy of ICB therapy in patients with NSCLC and oncogenic drivers. The updated results of the ImmunoTarget cohort included 551 patients (most common oncogenic driver was KRAS [49% of patients], followed by EGFR [23%], BRAF [8%] and MET [7%]). In the total cohort the response rate was 19%, PFS 2.8 months and OS 16.1 months. Response rate in patients harbouring an EGFR mutation was 12%. Thus ICB therapy might be an option after failure of treatment with tyrosine kinase inhibitors. On the other hand outcome in ALK positive patients (*n* = 23) was poor. Even though the sample size was small the results indicate that chemotherapy might be superior compared to ICB after tyrosine kinase exhaustion. Results revealed that PD-L1 expression had an impact on PFS in patients treated with ICB harbouring an EGFR mutation or KRAS mutation.

The outcomes in patients treated with ICB are consistent with registration trials, but inferior to targeted therapies in genetic driven NSCLC.

## Therapies in genetic driven NSCLC

The highlight in EGFR mutation positive NSCLC were the outcome data of the ARCHER 1050 trial. In this study dacomitinib, a second-generation irreversible EGFR tyrosine kinase inhibitor, demonstrated a superior OS to gefitinib in first-line treatment [12]. Nevertheless very interesting combinational approaches in EGFR mutated patients were presented. Nakamura et al. presented OS data on the use of gefitinib combined with chemotherapy versus gefitinib monotherapy in EGFR mutated patients [13]. This study showed that in EGFR mutation positive NSCLC the addition of carboplatin and pemetrexed to gefitinib significantly improved PFS and OS. The study has to be evaluated with some caveats as the combination therapy failed to demonstrate its superiority in prolongation of the second PFS (PFS2). However, it may result in an increase of long survivors as presented by prolongation of OS in the entire cohort.

Next the approach of combining EGFR TKI with anti-angiogenic therapy was investigated in two phase III studies (NEJ026 and JO25567): both studies showed that bevacizumab and erlotinib combination improved PFS compared to erlotinib monotherapy without increasing the rate of serious adverse events [14, 15].

## Conclusion

**Fig. 1:**
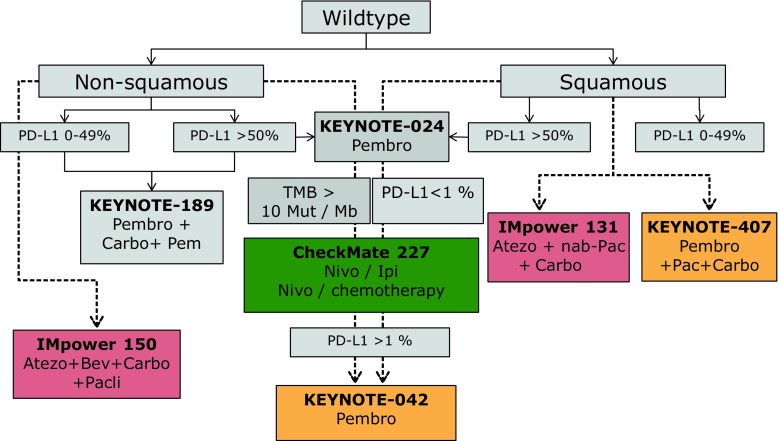
Integration of the most important phase III studies presented at ASCO 2018 in a possible therapy algorithm in advanced stage wildtype NSCLC. *Pembro* pembrolizumab, *PD-L1* programmed death-ligand 1, *Carbo* carboplatin, *Pem* pemetrexed, *Atezo* atezolizumab, *Bev* bevacizumab, *Pacli* paclitaxel, *TMB0* tumor mutational burden, *Mut/Mb* mutations per megabase, *Nivo* nivolumab, *Ipi* ipilimumab, *nab-Pac* nab-paclitaxel

ASCO 2018 NSCLC highlights were dominated by combination therapy approaches. The first-line developments in wild type NSCLC have to be evaluated in the light of two important studies (KEYNOTE-189, CheckMate 227) previously presented at the AACR meeting this year, which prepared already the stage for the presentations at ASCO 2018 [9, 16]. Four important ICB chemotherapy studies [4–7] have been presented and these combination strategies are practice changing and will lead to adapted therapy guidelines in the near future. Nevertheless the addition of ICB to chemotherapy backbones increases toxicity (grade 3–4 toxicity overall about 10% higher) when compared to chemotherapy alone and poses limited follow-up treatment options. Hence more data are needed and patient selection might be key for these strategies. We summarized the practice changing potential of these key studies within future treatment algorithms (Fig. 1).

In contrast to the main topic of ASCO 2018 “precision medicine” most trials showed significant benefit across all subgroups independent of the PD-L1 expression. Nevertheless higher PD-L1 was associated with better therapy response and TMB was further evaluated as a good biomarker for ICB efficacy especially in PD-L1 negative patients. Future biomarker research is ongoing and might be a multimodal approach combining different techniques (as often depicted in the cancer-immunogram [17]). TMB has also been shown to be evaluable on cfDNA in blood tests, guaranteeing broad and easy accessible sampling [18]. Next cfDNA also poses a source for NSCLC screening with highly sophisticated genetic tools [1].

In conclusion, multimodal biomarkers combinations (e.g. TMB, PD-L1, blood-based tests), new genetics tools for NSCLC screening and successful developments of ICB combinational strategies (either with chemotherapy, SBRT, anti-angiogenic agents or ICB) were the highlights presented at ASCO 2018.
